# Oxygen administration for postoperative surgical patients: a narrative review

**DOI:** 10.1186/s40560-020-00498-5

**Published:** 2020-10-14

**Authors:** Satoshi Suzuki

**Affiliations:** Department of Intensive Care, Okayama University Hospital, 2-5-1, Shikata-cho, Kita-ku, Okayama, 700-8558 Japan

**Keywords:** Oxygen therapy, Hyperoxemia, Hypoxemia, Postoperative care, Surgical site infection

## Abstract

Most postoperative surgical patients routinely receive supplemental oxygen therapy to prevent the potential development of hypoxemia due to incomplete lung re-expansion, reduced chest wall, and diaphragmatic activity caused by surgical site pain, consequences of hemodynamic impairment, and residual effects of anesthetic drugs (most notably residual neuromuscular blockade), which may result in atelectasis, ventilation–perfusion mismatch, alveolar hypoventilation, and impaired upper airway patency. Additionally, the World Health Organization guidelines for reducing surgical site infection have recommended the perioperative administration of high-dose oxygen, including during the immediate postoperative period. However, supplemental oxygen and hyperoxemia also have harmful effects on the respiratory and cardiovascular systems, with several clinical studies having reported an association between high perioperative oxygen administration and worse clinical outcomes. Recently, the increased availability of new and short-acting anesthetic drugs, comprehensive pharmacological knowledge, postoperative multimodal analgesia, and new minimally invasive surgery options could result in lower incidences of postoperative hypoxemia. Moreover, recommendations promoting high oxygen administration to prevent surgical site infections have been challenged, considering the lack of scientific investigations, and have not been widely accepted. Given the potential harmful effects of hyperoxemia, routine postoperative oxygen administration might not be recommended. Recent clinical studies have indicated that a conservative approach to oxygen therapy, where oxygen administration is titrated to achieve slightly lower oxygen levels than usual, could be safely implemented and decrease acutely ill patients’ susceptibility to hyperoxemia. Based on current evidence, appropriate monitoring, including peripheral oxygen saturation, and oxygen titration should be required during postoperative oxygen administration to avoid both hypoxemia and hyperoxemia. Future trials should therefore focus on determining the optimal oxygen target during postoperative care.

## Background

Most postoperative surgical patients routinely receive supplemental oxygen therapy to prevent hypoxemia, one of the most frequent and important postoperative respiratory complications. Moreover, while routine supplemental oxygen administration is not recommended for non-hypoxemic patients with several acute conditions, such as acute coronary syndrome [1, 2] and stroke [3, 4], the postoperative period is one of the few circumstances where high-dose oxygen therapy has been recommended to reduce surgical site infections (SSIs) [5]. However, such recommendations remain controversial and have not been widely accepted in actual clinical practice [6–8]. The present paper therefore reviews the rationale, clinical significance, and controversies regarding postoperative oxygen therapy and re-evaluates the optimal use of postoperative oxygen.

## Rationale for postoperative oxygen administration

Postoperative patients are susceptible to hypoxemia because of incomplete lung re-expansion, reduced chest wall, and diaphragmatic activity caused by surgical injury and pain, consequences of hemodynamic impairment, and residual effects of anesthetic drugs (most notably residual neuromuscular blockade) [9, 10], which may result in atelectasis, ventilation–perfusion mismatch, alveolar hypoventilation, and impaired upper airway patency among others (Table 1) [11]. Postoperative shivering may also increase oxygen consumption, thereby increasing the risk of hypoxemia [12]. Accordingly, prolonged hypoxemia may promote serious consequences, including arrhythmias [13, 14], myocardial ischemia [15], and cognitive dysfunction [16].

**Table 1:** Possible mechanisms of postoperative hypoxemia

Mechanism	Factors involved
Atelectasis	Direct compression of the lungs during operation, Incomplete lung re-expansion, Reduced chest wall and diaphragmatic movement due to surgical injury or pain
Alveolar hypoventilation	Depressed level of consciousness due to residual effect of anesthetic drugs, Postoperative opioids
Impaired upper airway patency	Residual effect of neuromuscular blocking agents, opioids, volatile anesthetics, or other agents with sedative properties, Airway tissue edema
Increased oxygen consumption	Shivering, Fever, Systemic inflammatory response

Reports have also shown that systemic hypoxemia impairs wound healing and suppresses certain aspects of the immune response, which can increase the risk of wound infections. Oxidative killing by neutrophils was impaired at low oxygen tensions often found in wounds [17]. Evidence therefore suggests that postoperative hypoxemia carries a significant risk and should be carefully avoided and immediately corrected. Accordingly, supplemental oxygen administration could increase oxygen tensions in the arterial blood, which might provide a safety buffer and prevent hypoxemic events at the cellular level.

## Possible harmful effects of postoperative hyperoxemic oxygen therapy

Oxygen has also been known to promote harmful effects. Indeed, hyperoxemia and excessive oxygen administration have a number of respiratory and cardiovascular effects. The lungs are particularly vulnerable to damage caused by prolonged exposure to high oxygen levels. Accordingly, oxygen breathing has been found to induce absorption atelectasis [18], while high fraction of inspired oxygen (FIO_2_) can impede minute ventilation in spontaneously breathing subjects, worsen ventilation–perfusion matching by countering hypoxic pulmonary vasoconstriction, and shift the carbon dioxide dissociation curve to the right (Haldane effect) [19]. Another potentially harmful consequence of administering unnecessarily high oxygen concentrations is impaired detection of hypoventilation via pulse oximetry. Supplemental oxygen in patients with spontaneous ventilation has been found to potentially promote delayed recognition of deteriorating lung function in the post-anesthesia care unit (PACU) [20].

Studies have shown that hyperoxemia reduces cardiac output by approximately 10% and increases systemic vascular resistance by 11–12% in both healthy volunteers and patients with coronary artery disease, while patients with heart failure exhibited a 15% decrease in cardiac output and a 25% increase in systemic vascular resistance [21]. Moreover, among patients undergoing elective coronary angiography, those who received 100% oxygen breathing had 20% lower coronary blood flow velocity and 23% higher coronary resistance relative to those who breathed room air [22].

Thus, some controversy exists regarding “routine” supplemental oxygen administration following surgery. However, the clinical significance of such potentially harmful effects during the postoperative period has remained uncertain [23].

## Prevention of postoperative hypoxemia

Hypoxemia has remained one of the most frequent and important postoperative respiratory complications. Accordingly, 35% of patients who underwent elective surgery under general anesthesia developed hypoxemia [peripheral oxygen saturation (SpO_2_) ≤ 90%] during postoperative transfer, with 12% developing severe hypoxemia (SpO_2_ ≤ 85%) [24]. An observational study by Sun et al., who analyzed oxygen saturation recorded at 1-min intervals for up to 48 h after the surgery in 833 patients undergoing noncardiac surgery, found that 21% and 8% of the patients had SpO_2_ levels of < 90% for longer than 10 and 20 min per hour, respectively [25]. In addition, 37% of patients experienced at least one episode where SpO_2_ was < 90% for an hour or more. Thus, postoperative hypoxemia is relatively common and prolonged.

Several studies have shown that supplemental postoperative oxygen may prevent hypoxemia. Accordingly, one study showed that although 19% of patients who were transferred without oxygen experienced initial oxygen desaturation (SpO_2_ < 90%) upon arrival at the PACU, only 0.8% of those with oxygen supplementation experienced the same [26]. Similar findings had also been reported by Maity et al. [27] who showed that 28% of patients who did not receive oxygen developed hypoxemia during the transfer from the operation theater to the recovery room, whereas none of those who received supplemental oxygen developed early postoperative hypoxemia. Therefore, routine oxygen administration during the immediate postoperative period could provide a margin of safety against hypoxemia and has been widely advocated for many years.

Although early studies have reported high incidences of postoperative hypoxemia (as high as 55%) [28], incidences have decreased to around 20% by the mid-2010s [25–27, 29]. Such a decrease might have been related to the increased availability of new and short-acting anesthetic drugs [30, 31], comprehensive pharmacological knowledge, and multimodal analgesia techniques for maximizing postoperative pain relief while minimizing side effects, and new minimally invasive surgery options. Thus, routine supplemental oxygen administration to prevent hypoxemia might not be necessary in most postoperative patients in recent years. In fact, a before-and-after comparison study showed that the shift from routine postoperative supplemental oxygen administration to titrated postoperative oxygen administration to maintain SpO_2_ levels at 90% or higher could increase the number of patients not needing supplemental oxygen therapy [32].

## Prevention of surgical site infection

In 2016, the World Health Organization (WHO) published recommendations for reducing SSIs. The panel performed a systematic review that compared the effects of perioperative administration of high FIO_2_ (80%) and standard FIO_2_ (30–35%) for the prevention of SSIs. Although their results showed that high FIO_2_ had no overall significant benefit on preventing SSI [odds ratio (OR) 0.84; 95% confidence interval (CI) 0.66–1.06], a subgroup analysis of trials where patients received oxygen therapy through a tracheal tube showed that increased perioperative FIO_2_ promoted a better reduction in SSI compared to standard perioperative FIO_2_ (OR 0.72; 95% CI 0.55–0.94) [33]. Based on the aforementioned evidence, the WHO recommended that adult patients undergoing general anesthesia with endotracheal intubation for surgical procedures should receive 80% FIO_2_ intraoperatively and, if feasible, for 2–6 h immediately after surgery. However, this has generated considerable controversy, especially regarding trial selection, statistical analysis [7, 34], and insufficient analysis of potentially negative systemic effects of high oxygen administration [35]. Additionally, one study included in the systematic review, which found that liberal oxygen use reduced the risk of infection after surgery, was retracted due to disputed credibility [36]. In their 2018 update, the WHO re-assessed the evidence, excluding questionable studies and including three new randomized controlled trials (RCTs) [37, 38]. Although the updated evidence showed that high FIO_2_ had weaker benefits and the new guidelines downgraded the strength of the recommendation from strong to conditional, the general recommendation to ventilate intubated surgical patients with an FIO_2_ of 0.8 was retained [5].

Nonetheless, the new recommendation has still been challenged [8, 39]. A recent large RCT including 717 patients undergoing abdominal surgery failed to identify any beneficial effect of high perioperative FIO_2_ on SSI [40]. Two recent studies, one being a retrospective analysis on administrative data from almost 74,000 patients undergoing non-cardiothoracic surgery [41] and the other being a prospective intervention study with more than 5700 patients undergoing intestinal surgery [42], had also generated controversy. Accordingly, both studies found that high FIO_2_ had no benefit on wound complications, with the retrospective study even finding a dose-dependent increase in frequency of pulmonary complications with high FIO_2_ [41]. Additionally, follow-up studies of the PROXI trial [43], one of the largest RCTs with the lowest possible risk of bias and arguably the best designed trial conducted to date, found that 80% oxygen administration during the perioperative period was associated with significantly increased long-term mortality [44], shorter time to cancer recurrence or death [45], and long-term risk of myocardial infarction [46]. Thus, concerns regarding high FIO_2_ and maintenance of hyperoxemia during and after surgery have still persisted [8, 23, 39].

## Re-evaluation of postoperative oxygen therapy

Based on the aforementioned discrepancy, routine postoperative oxygen administration and the recommendation of high perioperative FIO_2_ to reduce SSI need to be reconsidered. Thus, the goals of postoperative oxygen therapy should be to maintain normoxemia and avoid unnecessary oxygen administration.

To evaluate a patient’s oxygenation status, the SpO_2_ sensor could be the most appropriate device because it allows noninvasive and continuous monitoring; moreover, pulse oximetry has been ubiquitously used in hospitals worldwide. However, although SpO_2_ monitoring facilitates hypoxemia detection, it may not help adequately detect hyperoxemia because elevated PaO_2_ is not recognized once SpO_2_ is ≥ 98%. The oxygen reserve index (ORI), a novel, noninvasive, and continuous variable derived from the SpO_2_ signal, correlates to elevated PaO_2_ and can help detect hyperoxemia [47]. Reportedly, the use of ORI monitoring to titrate oxygen administration led to a favorable reduction in the time spent with hyperoxemia compared with the use of SpO_2_ alone in critically ill patients [48]. Such a new monitoring methodology might help improve postoperative oxygen therapy.

In addition, reports have shown that titration of supplemental oxygen to achieve targeted oxygen saturation could reduce oxygen exposure [49, 50]. Recently, two large RCTs comparing two different oxygen targets in critically ill patients, including those who had undergone surgery, have been published. In a single-center RCT in Italy, including a total of 434 patients, of which 62% were surgical patients, Girardis et al. compared conservative (target partial pressure of oxygen of 70–100 mmHg or SpO_2_ of 94–98%) with conventional (target partial pressure of oxygen of < 150 mmHg or SpO_2_ of 97–100%) oxygen therapy [51]. Although their findings showed that conservative oxygen reduced mortality rates, shock, liver failure, and bacteremia, their study suffered from serious methodological flaws, including a single-center design, baseline imbalances, and early termination after an unplanned interim analysis. Moreover, ICU-ROX, the largest RCT to date with 1000 patients, found no difference in outcomes between conservative (target SpO_2_ of 90–97%) and conventional (target SpO_2_ of 90% with no upper limit) oxygen therapy [52]. Overall, this study included 295 surgical patients (approximately 30% of their patients), which limits its postoperative care comparability. To date, no RCT comparing liberal (conventional) and conservative oxygen therapy has focused on postoperative patients. To our knowledge, only one study, uncontrolled before-and-after design, has evaluated the introduction of conservative oxygen therapy (target SpO_2_ of 88–92%) for cardiac surgical patients admitted to the intensive care unit [53]. Accordingly, this study found that conservative oxygen therapy could promote a significant decrease in hyperoxemia exposure without increasing the incidence of severe hypoxemia. Although such findings might support the feasibility and physiological safety of conservative oxygen therapy in patients admitted to the intensive care unit after surgery, currently available studies do not provide sufficient evidence to guide practice regarding postoperative oxygen therapy. In addition, there might be a discrepancy in oxygen levels defined as liberal oxygen therapy between the clinical studies comparing two different oxygen targets [51–53] and in the levels reported to exert physiological harmful effects of supplemental oxygen and hyperoxemia [18, 21, 22]. So far, no clinical studies have examined the threshold for the onset of the association of hyperoxemia or hypoxemia with poor outcomes in patients postoperatively. Thus, the safe levels of oxygen and optimal oxygen target in postoperative patients remain uncertain.

Despite the lack of high-quality evidence, the current guideline for oxygen use strongly recommends that oxygen saturation be maintained no higher than 96% for most patients in acute care settings, including postoperative patients [54]. Accordingly, until more evidence is available, hyperoxemia should not be routinely recommended, while unnecessarily high FIO_2_ should be avoided to maintain normoxemia during the postoperative period.

Although previous studies have compared different levels of FIO_2_, limited information regarding the number of patients who developed hypoxemia or hyperoxemia has been available. Therefore, such an oxygen dose might be insufficient for some patients but excessive for others. Future trials should therefore focus on oxygen titration to achieve target oxygenation levels in order to avoid both hypoxemia and hyperoxemia.

## Conclusions

Postoperative oxygen has been routinely administered to prevent hypoxemia, with the current guidelines recommending high perioperative oxygen administration to prevent SSIs. However, available evidence has highlighted the risks of the current practice and recommendations. In accordance with the “Do No Harm” principle, routine oxygen supplementation and unnecessary hyperoxemia should be avoided during postoperative settings. As such, appropriate monitoring, including SpO_2_, and oxygen titration should be performed during oxygen administration to maintain normoxemia. Future trials should therefore focus on determining the optimal oxygen target during postoperative care.

## Data Availability

Not applicable

## Abbreviations

FIO_2_: High fraction of inspired oxygen
PACU: Post-anesthesia care unit
SpO_2_: Peripheral oxygen saturation
WHO: World Health Organization
SSI: Surgical site infection
OR: Odds ratio
ORI: Oxygen reserve index
CI: Confidence interval
RCT: Randomized controlled trial
